# Transcriptional Control of Gene Expression and the Heterogeneous Cellular Identity of Erythroblastic Island Macrophages

**DOI:** 10.3389/fgene.2021.756028

**Published:** 2021-11-22

**Authors:** Kaustav Mukherjee, James J. Bieker

**Affiliations:** ^1^ Department of Cell, Developmental, and Regenerative Biology, Mount Sinai School of Medicine, New York, NY, United States; ^2^ Black Family Stem Cell Institute, Mount Sinai School of Medicine, New York, NY, United States; ^3^ Tisch Cancer Center, Mount Sinai School of Medicine, New York, NY, United States; ^4^ Mindich Child Health and Development Institute, Mount Sinai School of Medicine, New York, NY, United States

**Keywords:** erythroblastic island, mammalian hematopoiesis, macrophage identity, erythropoiesis, transcriptional control, single cell analyses, EKLF/KLF1

## Abstract

During definitive erythropoiesis, maturation of erythroid progenitors into enucleated reticulocytes requires the erythroblastic island (EBI) niche comprising a central macrophage attached to differentiating erythroid progenitors. Normally, the macrophage provides a nurturing environment for maturation of erythroid cells. Its critical physiologic importance entails aiding in recovery from anemic insults, such as systemic stress or acquired disease. Considerable interest in characterizing the central macrophage of the island niche led to the identification of putative cell surface markers enriched in island macrophages, enabling isolation and characterization. Recent studies focus on bulk and single cell transcriptomics of the island macrophage during adult steady-state erythropoiesis and embryonic erythropoiesis. They reveal that the island macrophage is a distinct cell type but with widespread cellular heterogeneity, likely suggesting distinct developmental origins and biological function. These studies have also uncovered transcriptional programs that drive gene expression in the island macrophage. Strikingly, the master erythroid regulator EKLF/Klf1 seems to also play a major role in specifying gene expression in island macrophages, including a putative EKLF/Klf1-dependent transcription circuit. Our present review and analysis of mouse single cell genetic patterns suggest novel expression characteristics that will enable a clear enrichment of EBI subtypes and resolution of island macrophage heterogeneity. Specifically, the discovery of markers such as Epor, and specific features for EKLF/Klf1-expressing island macrophages such as Sptb and Add2, or for SpiC-expressing island macrophage such as Timd4, or for Maf/Nr1h3-expressing island macrophage such as Vcam1, opens exciting possibilities for further characterization of these unique macrophage cell types in the context of their critical developmental function.

## Introduction

The erythroblastic island (EBI) niche is one of the earliest observed and described stem cell niches in biology consisting of a central macrophage that is attached to erythroid cells at various stages of differentiation (Bessis, 1958; Mohandas and Prenant, 1978; Bernard, 1991). This macrophage aids erythroid maturation by providing cytokines and growth factors, enables enucleation by providing phagocytic functions, and ultimately provides an effective means for reticulocyte formation and release (reviewed comprehensively in Chasis and Mohandas (2008), Manwani and Bieker (2008), de Back et al. (2014), Hom et al. (2015), Klei et al. (2017), Li et al. (2020)). As a result, considerable effort has been dedicated to characterizing EBI macrophages by studying cell surface marker expression for their efficient isolation (Soni et al., 2006; Porcu et al., 2011; Chow et al., 2013; Seu et al., 2017; Li et al., 2019), with various groups reporting different combinations of markers associated with them (Sadahira et al., 1991; Sadahira et al., 1995; Chow et al., 2013; Seu et al., 2017; Li et al., 2019; Yeo et al., 2019b; Mukherjee et al., 2021; Zhang et al., 2021).

These studies however reveal that EBI macrophages are inherently composed of heterogeneous subpopulations, and distinct subtypes of EBIs have also been reported (Seu et al., 2017; Tay et al., 2020; Zhang et al., 2021). Surprisingly, despite their seemingly crucial role in *in vivo* erythropoiesis, depletion of EBI macrophages does not affect steady-state erythropoiesis (Korolnek and Hamza, 2015; Ulyanova et al., 2016). However, EBI alterations or defects during steady-state erythropoiesis may not necessarily be manifest as severe phenotypes because adequate coping mechanisms to recover and repair EBIs are only required during stress. This is supported by the fact that EBI macrophages are critical for recovery from stress erythropoiesis in the splenic red pulp (Sadahira et al., 2000; Chow et al., 2013; Ramos et al., 2013; Jacobsen et al., 2015; Liao et al., 2018; May and Forrester, 2020). Recovery is effectively aided by *de novo* differentiation of monocytes into macrophages (Ulyanova et al., 2016; Liao et al., 2018) and via epo-induced signaling in island macrophage that yields prostaglandin mediators of stress erythroid progenitors (Chen et al., 2020). Clinical reports of altered EBI structures correlating with disease have been observed, particularly after examining the effect of primary myelofibrosis (PMF) on the bone marrow (BM) or of lower than normal macrophage levels in some acute leukemias (Koury, 2014). Modulating the effects of ß-thalassemia and polycythemia vera (PV) by targeting the central macrophage have been proposed (Li et al., 2020). Additionally, a significant proportion of myelodysplastic syndrome (MDS) patients have alterations in EBIs, particularly of numerical density and size, that correlate with severe anemia and poor disease prognosis independent of other factors such as age (Buesche et al., 2016).

Until recently, detailed characterizations of EBI macrophage heterogeneity and function were hampered by a lack of global gene expression profiles in these macrophages. This was partly due to uncertainty regarding cell surface markers that would specifically enrich for the subpopulation of macrophages that form the EBI niche (Seu et al., 2017; Tay et al., 2020). F4/80 antigen expression is associated with EBI macrophages (Sadahira et al., 1991; Manwani and Bieker, 2008) although not all F4/80+ macrophages in hematopoietic tissues necessarily form EBIs. Nevertheless, recent studies have used F4/80 in addition to surrogate markers such as pEKLF/GFP or Epor-eGFP to enrich for EBI macrophage subpopulations from primary mouse hematopoietic tissue and have determined global gene expression at the bulk (Xue et al., 2014; Li et al., 2019; Mukherjee et al., 2021) and single cell level (Mukherjee et al., 2021). These studies have uncovered novel aspects of EBI macrophage identity, heterogeneity, and physiological function while reinforcing certain existing paradigms. They have also shed substantial light on some unanswered questions regarding transcriptional control of gene expression in EBI macrophages.

The goal of this review is to summarize the findings of the bulk transcriptomic studies of murine EBI macrophages, and to delve deeper into the single cell RNA-Seq data to reveal new aspects of cellular identity and heterogeneity, and the underlying transcription programs putatively controlling gene expression in EBI macrophages.

## The Transcriptomics of Epor+ EBI Macrophages

Two compelling sets of observations led to the hypothesis that the erythropoietin receptor (Epor) is expressed in EBI macrophages and EPO signaling is important for EBI macrophage function. First, EPO signaling and addition of recombinant human EPO had been noted to increase the expansion and activity of the erythroid and macrophage compartments in mice primary tissue (Lifshitz et al., 2010; Xiang et al., 2015; Luo et al., 2016; Wang et al., 2018). Second, Epor expression is not restricted to erythroid cells and is indeed expressed in wide variety of tissues (discussed in Zhang et al. (2021)). Based on these studies it was hypothesized and demonstrated that BM F4/80+ macrophages express Epor, and that the fraction of F4/80+ macrophages that form EBIs is significantly enriched for Epor+ macrophages, thus establishing a positive correlation between Epor expression in macrophages and propensity for EBI formation (Li et al., 2019). Thus, Epor was established as a novel marker for EBI-enriched macrophages in combination with F4/80 (Paulson, 2019).

This enabled the development of a strategy to use Epor as a marker for isolating EBI macrophages with an Epor-/eGFPcre knock-in mouse. BM F4/80+Epor/eGFP+ and F4/80+Epor/eGFP- populations (also Ter119-Ly6G-, thus not inclusive of erythroid cells or granulocytes) were separated by FACS and global gene expression in the two sub-populations compared by bulk RNA-Seq (Li et al., 2019). As expected, the two populations show inherently distinct gene expression signatures, with the Epor/eGFP+ macrophages expressing genes specifically involved in EBI niche functions such as iron homeostasis (*Hmox1*, *Slc40a1*, *Tfrc*), cell adhesion (*Vcam1*, *Siglec1*), phagocytosis and nuclear engulfment (*Mertk*, *Tim4*). In contrast, the Epor/eGFP- macrophages express innate immunity genes involved in functions such as inflammation, macrophage activation, and activation of the adaptive immune response (Li et al., 2019). Notably, a few transcription factors including EKLF/Klf1 and Spi-C are enriched in Epor/eGFP+ macrophages, indicating that transcription regulation in EBI macrophages depends on the action of these transcription factors. The liver X receptor α (*Nr1h3*), implicated in lipid metabolism in macrophages and inflammation (Zhao et al., 2021), and the transcription factor Maf, which has been shown to regulate F4/80 (*Adgre1*) and IL-10 expression in macrophages (Cao et al., 2005; Nakamura et al., 2009; Kusakabe et al., 2011), are also enriched in Epor+ macrophages. Elucidating their specific roles in regulating transcription in EBI macrophages is thus a promising avenue for further investigation.

## EKLF/Klf1 Expression and Function in FL F4/80+ Macrophages

Although EKLF/Klf1 is mostly known for its tissue-restricted, global transcriptional role in facilitating maturation and differentiation of proerythroblasts into mature reticulocytes (Tallack and Perkins, 2010; Siatecka and Bieker, 2011; Gnanapragasam and Bieker, 2017) and in fetal to adult globin switching (Bieker, 2010; Perkins et al., 2016), cellular analyses show that EKLF is also critical for the integrity of EB islands (Xue et al., 2014). Consequently, EBIs isolated from EKLF^−/−^ mouse fetal livers (FL) exhibit quite a few aberrations; for example, there are overall fewer F4/80+ macrophages (Mukherjee et al., 2021), and isolated EB islands have severely altered morphology compared to EKLF^+/+^ FL (Xue et al., 2014). Thus, our previous understanding of EKLF’s role in erythropoiesis as being restricted to the erythroid compartment required reassessment for which, firstly, EKLF expression in F4/80+ macrophages had to be directly and clearly demonstrated. This was accomplished by purifying F4/80+ macrophages from E14.5 FL and detecting EKLF mRNA expression by RT-PCR and protein expression by immunostaining (Porcu et al., 2011; Mukherjee et al., 2021). Subsequently, using a transgenic mouse expressing GFP under the EKLF promoter (pEKLF/GFP; (Lohmann and Bieker, 2008)) it was shown that almost 30% of all F4/80+ macrophages from E13.5 FL are GFP+ (and thus likely EKLF+) (Xue et al., 2014). Indirect evidence (Li et al., 2019) demonstrated EKLF mRNA enrichment in Epor+F4/80+ macrophages that are more likely to form EBIs, indicating that EKLF expression is associated with EBI-forming macrophages. A direct analysis of RNA shows transient expression of EKLF in staged, sorted macrophage cells from the developing mouse FL (Mass et al., 2016). Finally, single cell RNA-Seq as well as flow cytometry directly show that a subpopulation of about 25% of total F4/80+ cells in E13.5 mouse FL express EKLF mRNA and protein (Mukherjee et al., 2021).

Earlier studies suggested that EKLF is important for the expression of DNase2a and Vcam1 in EBI macrophages (Porcu et al., 2011; Xue et al., 2014). However, given the severe alterations in EBIs from EKLF^−/−^ mice, and the known global role of EKLF in erythroid cells, it was unlikely that EKLF function in EBI macrophages would be restricted to just these two genes. To address whether EKLF also plays a global role in specifying gene expression in EBI macrophages one has to consider that since not all EBI macrophages express EKLF, one cannot simply study FL F4/80+ macrophage gene expression in EKLF^−/−^ mice and derive definitive conclusions. An ideal system would be a conditional EKLF^−/−^ in F4/80+ macrophages, but such a genetic system is not available as yet. Thus, in our study (Mukherjee et al., 2021) we combined gene expression data from two separate mouse genetic models to address this question.

First, we used the pEKLF/GFP mouse (Lohmann and Bieker, 2008) to separate EKLF/GFP+ and EKLF/GFP- F4/80+ macrophages from E13.5 mouse fetal liver and showed that, similar to Epor/eGFP+, only the EKLF/GFP+ macrophages are enriched for genes whose functions are relevant to the EBI niche macrophages. This includes iron homeostasis and transport, heme metabolism and erythroid differentiation (Mukherjee et al., 2021). Among the genes enriched in EKLF/GFP+ macrophages, 99 genes are also enriched in Epor/eGFP+ BM macrophages (Supplementary Table S2) and GO analysis of this gene set shows that they only contain genes involved in heme metabolism and iron homeostasis (Supplementary Table S1). The two RNA-Seq datasets were generated from very different erythropoiesis environments (adult BM vs embryonic FL), and thus these analyses show that EKLF+/Epor+ macrophages play a crucial role in providing iron and heme to developing erythroblasts at early stages of development and adult steady state erythropoiesis (Korolnek and Hamza, 2015; Ganz, 2016; Alam et al., 2017; Yeo et al., 2019a).

Second, RNA-Seq on F4/80+ macrophages isolated from EKLF^+/+^ and EKLF^−/−^ E13.5 FL shows that the predominant effect on gene expression in the absence of EKLF is gene downregulation (Mukherjee et al., 2021). This is consistent with the major role of EKLF as a transcription activator via its interaction with histone acetylases and other chromatin modifiers (Yien and Bieker, 2013), and seemingly EKLF activates the transcription of specific targets in F4/80+ macrophages. Further, about 500 genes that are significantly downregulated in EKLF^−/−^ and enriched in EKLF/GFP+ macrophages are likely to be directly activated by EKLF binding to their promoters. This is supported by bioinformatic analysis showing that their promoters contain EKLF binding motifs (Mukherjee et al., 2021), but this in silico observation requires experimental validation. Binding motifs of other Klf family and E2f family TFs are also enriched in these promoters, and identification of these TFs here are reminiscent of a part of the EKLF-dependent transcriptional program in erythroid cells (Funnell et al., 2007; Eaton et al., 2008; Pilon et al., 2008; Tallack et al., 2009). This suggests the presence of either a transcription circuit or coregulators of transcription along with EKLF that may specify gene expression in EBI macrophages, but this again needs to be further validated by targeted genetic and molecular experiments to determine the specific roles of these TFs in EBI macrophages. In addition, other transcription factors in the EKLF-dependent gene expression program include MafK (NF-E2), Foxo3, Ikzf1, and Nr3c1(glucocorticoid receptor (Falchi et al., 2015)). It has been noted that KLF recognition motifs are enriched in active genes and are uniquely associated with CJUN binding in genetically diverse mouse macrophage (Gosselin et al., 2014; Lavin et al., 2014; Link et al., 2018). These findings point to a definitive global role for EKLF in EBI macrophages, distinct from its erythroid role, but towards the same goal of ensuring efficient *in vivo* erythropoiesis.

## Cellular Heterogeneity in E13.5 FL Macrophages Revealed by Single Cell RNA-Seq

The heterogeneity of macrophages in erythropoietic tissues has been apparent and efforts have attempted to resolve heterogeneity by assaying different cell surface markers associated with various subpopulations (Seu et al., 2017; Li et al., 2019; Tay et al., 2020). Even within the fraction of FL macrophages that form EBIs, there was evidence of heterogeneity due to distinct cell surface marker expression as well as varying morphology (Chasis and Mohandas, 2008; Manwani and Bieker, 2008; Yeo et al., 2016). Consistent with these studies, the Epor/eGFP and EKLF/GFP RNA-Seq datasets show at least two distinct populations in each case (Li et al., 2019; Mukherjee et al., 2021) but firm conclusions about the extent of heterogeneity are difficult to draw from bulk RNA-Seq data. Single cell RNA-Seq (sc-seq) has been developed precisely for this purpose, and most researchers in the field were eager to apply sc-seq on EBI macrophages to resolve heterogeneity.

**FIGURE 1:**
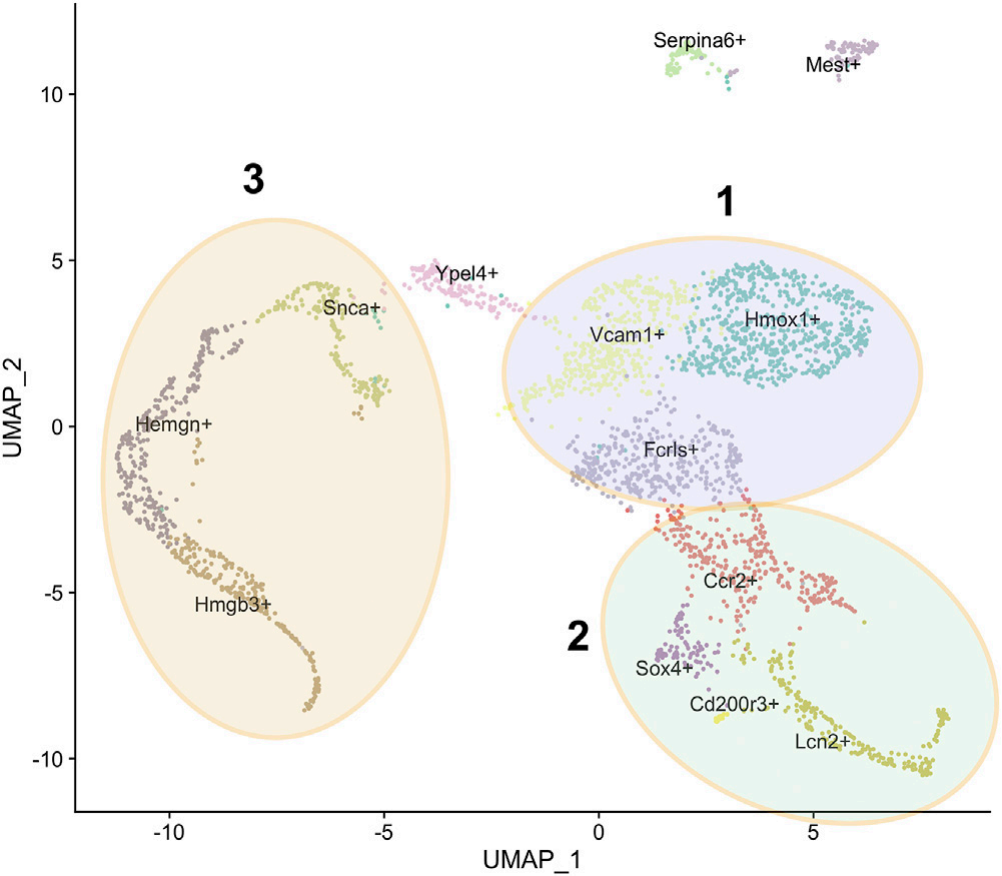
Subpopulations of F4/80+ macrophages from E13.5 mouse fetal liver. U-MAP clustering analysis of single cell RNA-Seq data from Mukherjee et al. (2021) showing the 13 subpopulations with the name of a unique marker for each subpopulation superimposed on it. Three superclusters are as indicated, based on GO analysis of highly expressed genes as described in the text: the Hmox1+, Vcam1+, Fcrls+ supercluster 1 in blue, the Ccr2+, Lcn2+, Sox4+ supercluster 2 in green, and the Snca+, Hemgn+, Hmgb3+ supercluster 3 in yellow.

Our group published sc-seq data on F4/80+ macrophages isolated from E13.5 mouse FL (Mukherjee et al., 2021). A challenge that still persists is to decide upon a reliable marker for isolating EBI macrophages among the fraction of total macrophages and study only that fraction. We opted to use F4/80, which is expressed on the surface of all macrophages, and performed an unbiased survey of the extent of their heterogeneity based on single cell gene expression profiles. We applied unsupervised clustering and allowed for the maximum resolution of clusters based on principal component analysis. Our FL sc-seq shows more than 10 subpopulations with distinct gene expression profiles in each subpopulation corresponding to distinct functions and cell types. Further each cluster is associated with unique expression of at least one marker (Figure 1). We will elaborate further on the nature of the major subpopulations in greater detail in the following sections based on this sc-seq data (Mukherjee et al., 2021).

## Cellular Properties, Marker Expression, and Functions of Various FL Macrophage Subpopulations

Most of the F4/80+ FL macrophages cluster near each other in the U-MAP of sc-seq data, apart from two small subpopulations marked by Serpina6 and Mest transcripts (Figure 1). All clusters express the *Adgre1* transcript that encodes the F4/80 protein, but each cluster expresses a variable amount (Mukherjee et al., 2021). This is consistent with the amount of F4/80 protein expression determined by Flow Cytometry that also shows variability in various cells. Based on GO analysis of the top significantly enriched genes in the minor Serpina6+ and Mest+ populations, it appears that the Serpina6+ population has enriched expression of genes involved in blood coagulation. Thus, these may be a small population of megakaryocyte lineage cells. Further, the Mest+ cluster likely comprises cells of mesodermal origin that are destined to form vasculature. Finally, the last subpopulation containing few cells is the Cd200r3+ population which comprise likely antigen-presenting macrophages that are enriched for genes involved in T-cell activation via antigen presentation.

**FIGURE 2:**
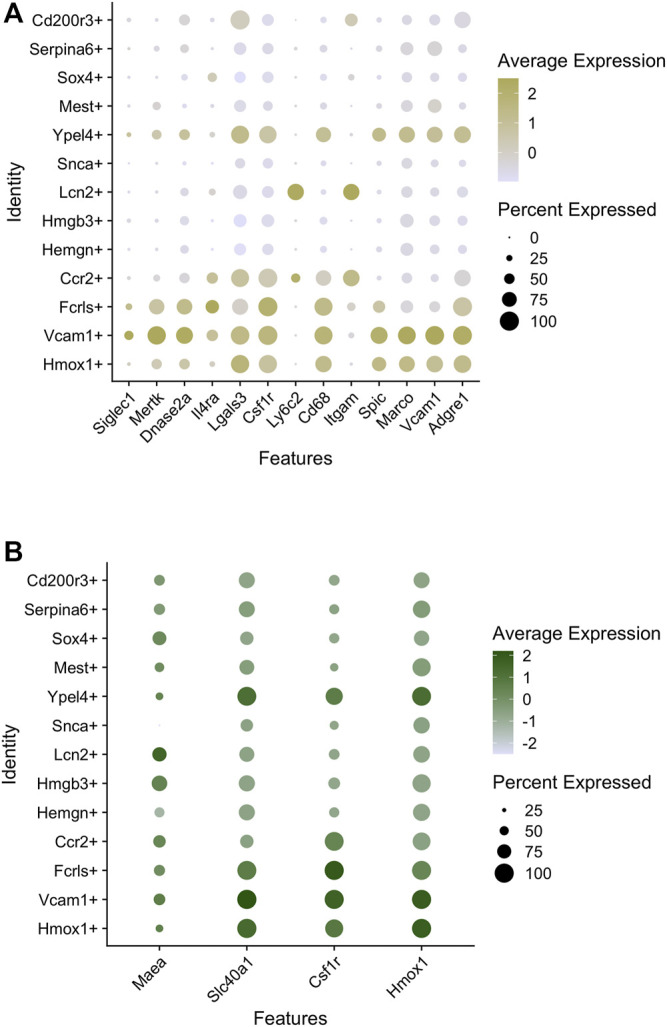
Macrophage marker expression in subpopulations of mouse E13.5 FL macrophages. **(A)** Bubble-plots showing the expression level of macrophage markers enriched in certain subpopulations. The intensity of the color indicates expression level of each gene whereas the size of the bubble depicts the relative fraction of cells in that subpopulation where the gene is expressed. **(B)** Bubble plots showing macrophage markers with similar levels of expression in most subpopulations.

Of the major populations, it is evident that there are three broad categories based on GO analysis of highly expressed genes. One comprises the Hmox1+, Vcam1+, Fcrls+ supercluster (Figure 1 - blue), second the Ccr2+, Lcn2+, Sox4+ supercluster (Figure 1 - green), and third the Snca+, Hemgn+, Hmgb3+ supercluster (Figure 1 - yellow). The Ypel4+ cluster has intermediate properties of superclusters 1 (Hmox1/Vcam1/Fcrls) and 3 (Snca/Hemgn/Hmgb3), as is also evidenced by its position in the U-MAP (Figure 1). Based on differentially expressed genes, the Hmox1+ and Vcam1+ subpopulations have a significant overlap in gene expression. These include endocytosis/cell import, cell adhesion, cell motility, iron homeostasis, cytoskeleton remodeling, innate immune response, myeloid leukocyte activation, cytokine production, and some metabolic processes. The Fcrls+ subpopulation also has similar properties as the Vcam1+ subpopulation with notable common functions such as MAPK signaling, ERK1/2 signaling, IL-1 and IL-6 production, which are important for erythroblast maturation. Thus, it appears that the Hmox1+, Vcam1+, Fcrls+ clusters are at least partly comprised of macrophages that form the EBI niche, consistent with the fact that both *Hmox1* and *Vcam1* are known to be expressed in EBI macrophages (Sadahira et al., 1995; Fraser et al., 2015). In fact, on surveying known macrophage markers (that may or may not correlate with EBI niche formation), one finds that although many markers have similar expression in the Vcam1+ and Hmox1+ subpopulations (Figure 2A), the Vcam1+ subpopulation has significantly enriched expression of *Dnase2a*, *Mertk*, *Siglec1 (Cd169)* and *Il4rα* compared to the Hmox1+ subpopulation. *Hmox1* expression itself is fairly ubiquitous in the isolated F4/80+ population, as is the expression of *Slc40a1 (ferroportin)* (Figure 2B)*,* suggesting that expression of these genes may not be solely restricted to EBI macrophages.

In contrast, supercluster 2 (Ccr2+/Lcn2+/Sox4+) is enriched for genes involved in innate immunity, T-cell activation, cytotoxicity, leukocyte and lymphocyte differentiation, T and B-cell development, suggesting that these subpopulations are similar to the Cd200r3 subpopulation also residing on the same arm of the U-MAP adjacent to these clusters. Therefore, these subpopulations of cells are likely comprised of activated macrophages that are geared towards provide innate immunity and assist in the development and differentiation of the adaptive immune response arm consisting of lymphocytes (Gautier et al., 2012; Lavin et al., 2014; Mass et al., 2016). One can rule out their involvement in forming the EBI niche.

The other arm of the U-MAP containing supercluster 3 (Snca+/Hemgn+/Hmgb3+) and the Ypel4+ subpopulation (Figure 1) is predominantly enriched for genes that are involved in erythroid and myeloid development, heme synthesis, and iron homeostasis, functions that are again attributable to the EBI niche (Korolnek and Hamza, 2015; Ganz, 2016; Alam et al., 2017; Yeo et al., 2019a). The Ypel4+ cluster is interesting since it not only expresses these genes, but also expresses genes involved in endocytosis, cell motility and cell adhesion indicating that this subpopulation of cells has some properties of the Hmox1+/Vcam1+ subpopulation as well. Indeed, this cluster is adjacent to the Vcam1+ cluster and expresses high levels of Vcam1 and Epor. The Hmgb3+ subpopulation seems to be composed of actively cycling cells with expression of genes involved in cell cycle, ribosome synthesis, protein synthesis, RNA processing and splicing. Supercluster 3 also express the EKLF/Klf1 transcription factor where Epor is partly enriched in the Snca+/Hemgn+ subpopulations. This is consistent with bulk RNA-seq data from our group as well as from Li et al. (2019) showing that EKLF+ macrophages are enriched for Epor expression and vice versa, but also demonstrating that there are unique subpopulations of EKLF and Epor expressing macrophages. It is tempting to speculate that the Hemgn+/Snca+ macrophage subpopulation expressing both EKLF and Epor form one subtype of EBI niche, whereas the Ypel4+/Vcam1+ subpopulations form another, but testing this hypothesis requires carefully crafted experiments.

**FIGURE 3:**
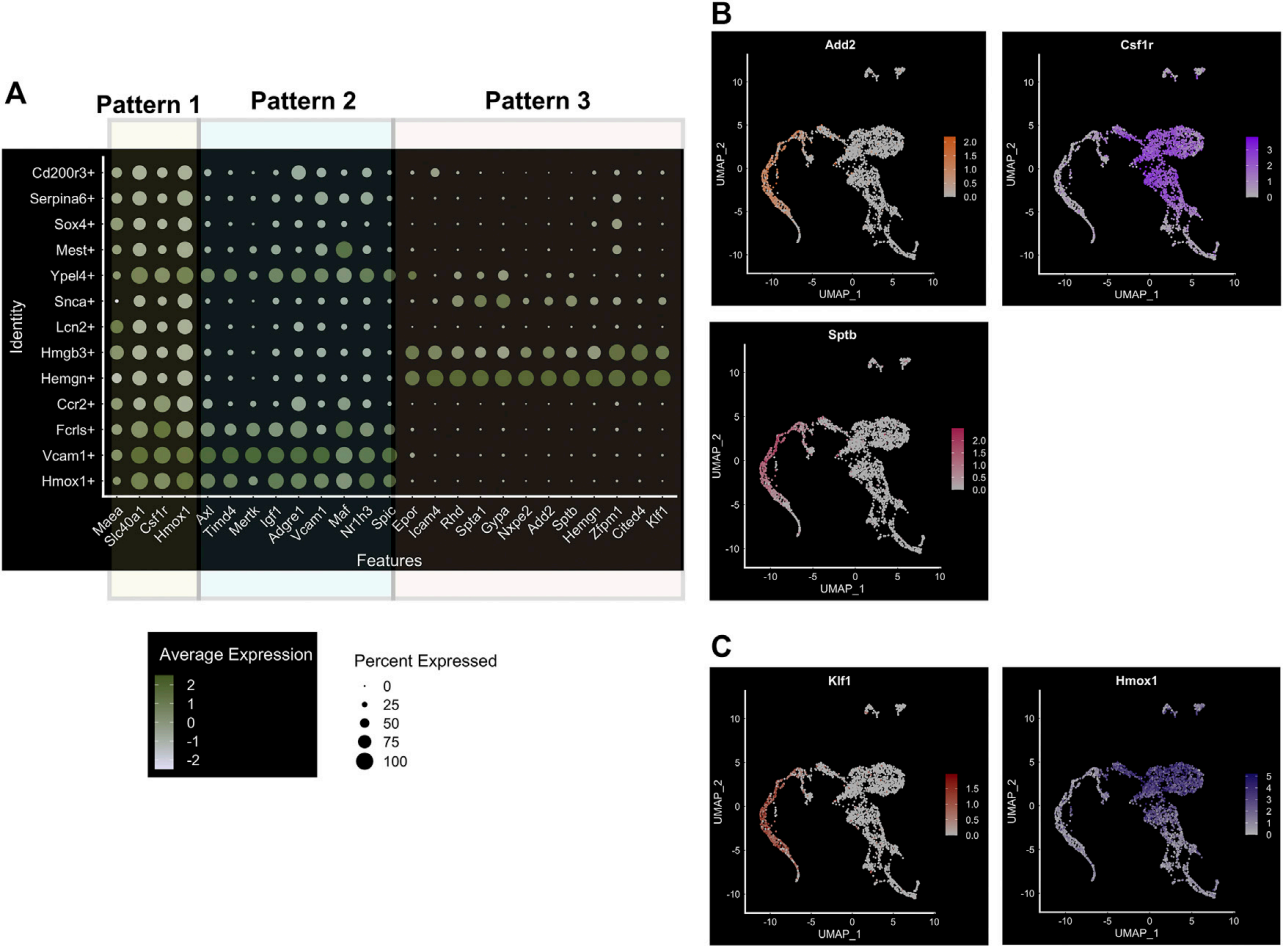
Major subpopulations of mouse E13.5 FL macrophages. **(A)** Bubble-plot showing specific genes expressed in the three major superclusters of FL macrophages, including population-specific transcription factors. Three patterns are segregated as indicated, based on their common expression as expanded upon in the text. **(B)** Feature plot showing cell-surface marker expression associated with the superclusters 3 and 1, *Sptb/Add2* and *Csf1r*, respectively. **(C)** Feature plot showing EKLF/*Klf1* expression; compare to positive overlap with *Sptb/Add2* in supercluster 3 (shown in **(B)**), but lack of overlap as compared to the cell-surface marker expression associated with supercluster 1, *Hmox1*.

On closer examination of the entire population for the expression of specific genes and cell surface markers of macrophages, we can detect 3 major patterns of marker expression (Figure 3A). We already found that *Hmox1*, *Fpn (Slc40a1)*, *Csf1r*, and *Maea* (“pattern 1”) are ubiquitously expressed in the whole population, and there is only a slight variation of expression in different subpopulations (Figure 3A). It would be interesting to determine the nature of this variability to determine cause-effect relationships between marker expression and heterogeneity. Additionally, we find that *Vcam1* expression correlates highly with *Axl*, *Timd4*, *Mertk*, and *Igf1* (“pattern 2”; Figure 3A), and all of these genes are known to be expressed in subtypes of EBI niches (e.g., MerTK (Toda et al., 2014)). Finally, the third pattern of marker expression includes *Epor*, *Gypa*, *Rhd*, *Icam4*, *Add2*, and *Sptb* (Figure 3A). An interesting dichotomy is revealed from this analysis when we compare the expression of *Add2/Sptb* with *Csf1r* and find that they are expressed in mutually distinct superclusters (Figure 3B). Interestingly, these superclusters also correlate with *EKLF/Klf1* and *Hmox1* expression respectively (Figure 3C). Perhaps this is indicative of the heterogeneity seen within EBI macrophages with respect to gene expression and morphological features such as domed and flat (Yeo et al., 2016; Yeo et al., 2019b). Whether this may be due to different developmental origins or different stages of erythroid maturation of attached erythroblasts remains to be explored.

## Specification of Heterogeneous Cell Identity of FL F4/80+ Macrophages by Key Transcription Factors

**FIGURE 4:**
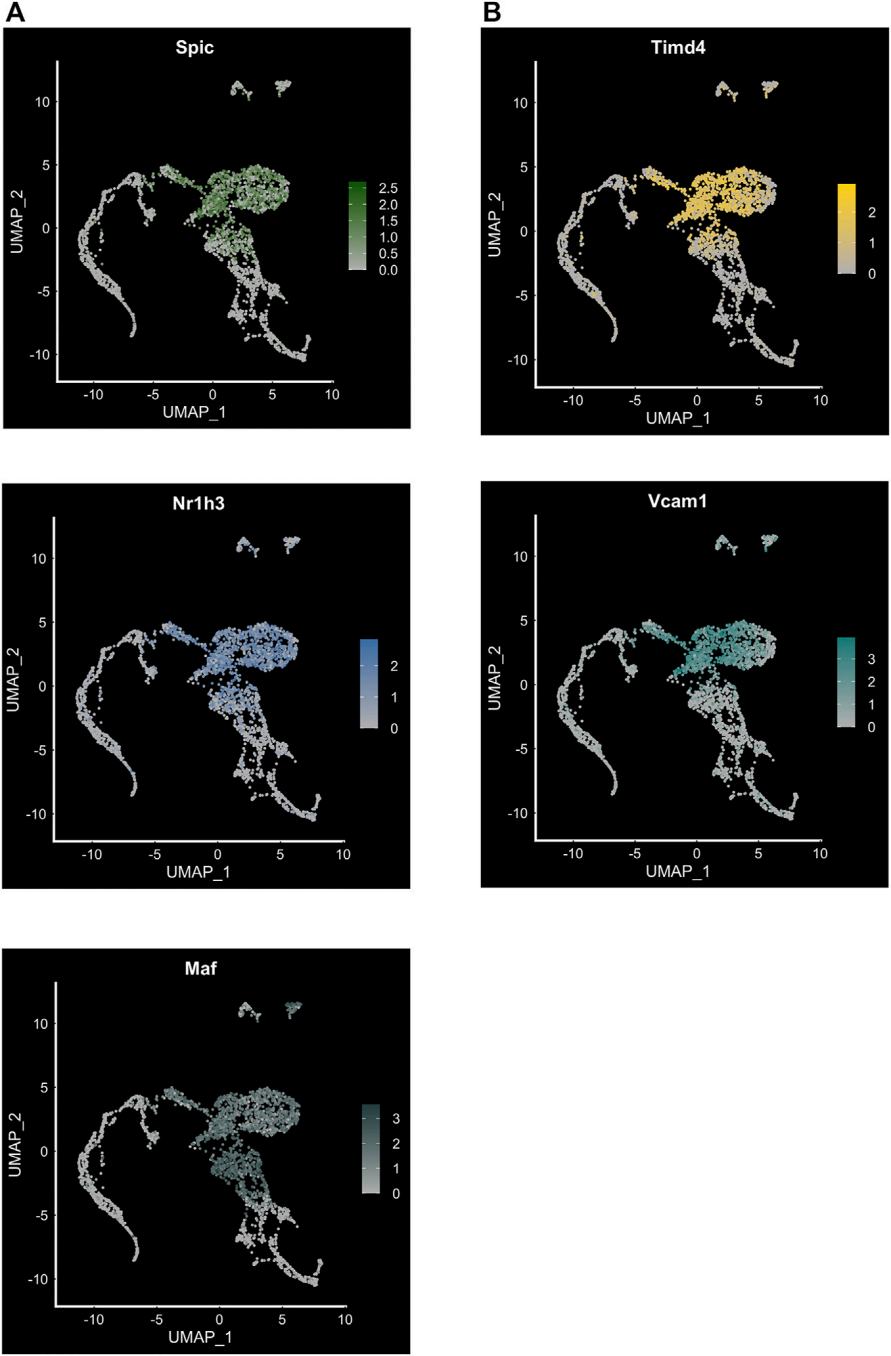
Transcription factor expression and cell surface markers in FL macrophage subpopulations. **(A)** Feature plot showing expression of transcription factors *Spic*, *Maf* and *Nr1h3* in the EKLF^−/−^ subpopulation of FL macrophages (compare to Figure 3C). **(B)** Feature plot showing expression of cell surface markers *Timd4* associated with *Spi-C*+, and *Vcam1* associated with *Maf+/Nr1h3*+ subpopulations.

Our study focused primarily on the role of EKLF/Klf1 in FL macrophages, and hence we looked for cell surface markers of the EKLF+ population, finding that *Add2* (Adducin-β), *Sptb* (β-spectrin), and *Nxpe2* mRNA expression correlates very highly with EKLF and shows the same transient expression as EKLF (Mukherjee et al., 2021). We also demonstrated that Adducin-β and β-spectrin proteins are expressed in a subpopulation of EBI macrophages. This confirms that the Hmgb3+/Hemgn+ subpopulations that are EKLF+ and Epor+ form at least one subtype of EBI macrophages. In addition, we find other cell surface markers enriched in the EKLF+ subpopulations but not correlating exclusively due to expression in the Ypel4 cluster, e.g., *Gypa*, *Epor*, *Spta1*, and *Rhd* (Figure 3A). The EKLF- subpopulations have enriched expression of the transcription factors Spi-C (*Spic*), *Maf*, and *Nr1h3* (Figures 3C and 4A) each of which are implicated in EBI macrophage function (Kohyama et al., 2009; Kusakabe et al., 2011; Haldar et al., 2014; Zhao et al., 2021). We find that *Klf1* and *Spic/Maf/Nr1h3* expression are mutually exclusive, fitting into patterns 3 and 2 respectively (Figure 3A). This suggests that these transcription factors drive transcription in distinct subpopulations of FL macrophages, and by extension, likely different categories of EBI macrophages. Exploring the direct transcriptional roles of EKLF, Spi-C, Maf and Nr1h3 could lead to interesting insights into whether these subpopulations are also distinct in terms of morphology, developmental origin, and function in the EBI niche. In the context of bulk RNA-Seq studies (Li et al., 2019), it may appear surprising that Epor+ macrophages are enriched for *EKLF/Klf1* as well as *Spic*, *Maf*, and *Nr1h3*, but this can be explained by the overlap in *Epor* and *Spic/Maf/Nr1h3* expression in the Ypel4+ cluster as well as the Epor+ EKLF+ supercluster (Hmgb3+/Hemgn+) (Figure 3A).

**FIGURE 5:**
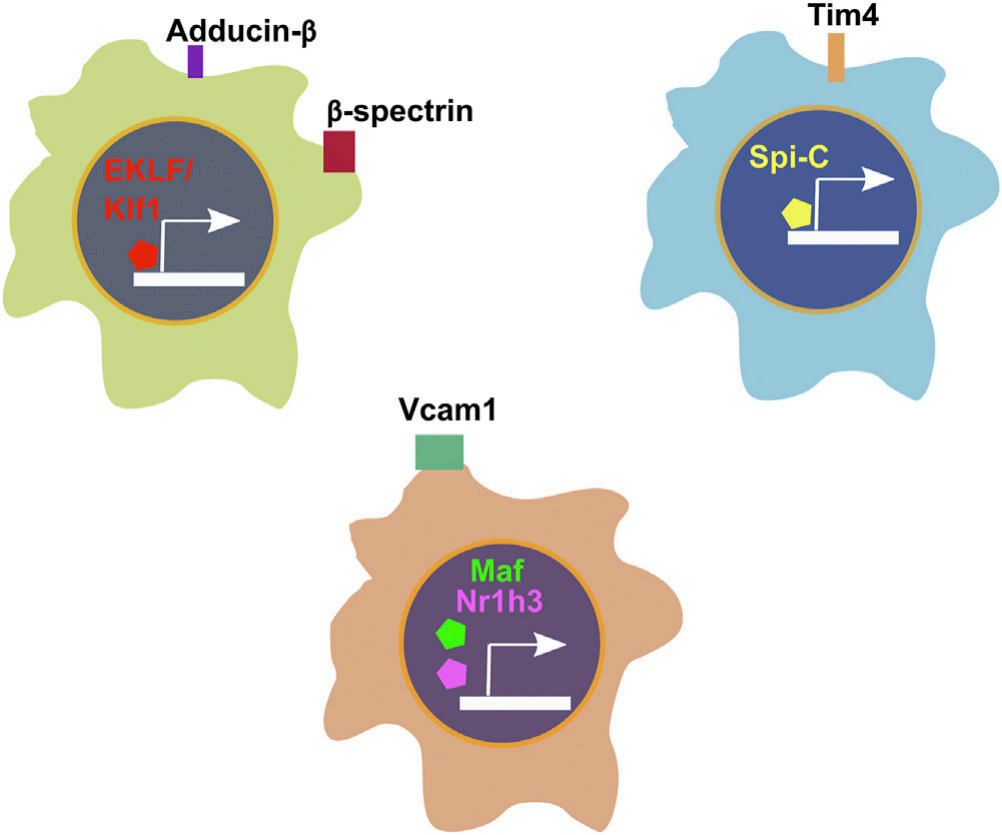
Summary of transcription factors and corresponding cell surface markers associated with cellular heterogeneity in E13.5 FL macrophages.

Similar to our approach for EKLF, we searched for cell surface markers that correlate with transcription factor expression in FL macrophages and find that *Spic* expression correlates highly with *Timd4* and that *Maf* and *Nr1h3* are most closely coexpressed with Vcam1 (Figures 4A and B)*.* Consequently, to develop a viable strategy for separating the EKLF and Spi-C subpopulations, one approach would be to use the cell surface markers *Sptb/Add2* and *Timd4* or *Vcam1* to separately isolate EKLF+ and Spi-C+/Maf+/Nr1h3+ subpopulations for detailed characterization (Figure 5). As alluded to earlier, it would be incredibly interesting to determine whether these starkly distinct populations have separate developmental origins but convergent functions in erythroid development. It is also likely that both subpopulations form EBI niches, but in different contexts depending on the stage of maturation of the attached erythroid progenitors.

## Summary and Outstanding Questions

From earlier studies focusing on morphology and cell surface marker expression in EBI macrophages in various hematopoietic tissues, to the current attempts in determining gene expression at bulk and single cell resolution in various tissues, the characterization of EBI macrophages have come a long way. However, there is much to learn about this intriguing cell-type in all the above areas. Despite earlier efforts, we still lack clarity on the exact nature and types of EBI macrophages based on cell surface marker expression, and as a result, we still lack a coherent strategy for isolation of various EBI macrophage subpopulations to study their form and function. Population heterogeneity continues to complicate interpretations of data generated using F4/80+ macrophages. In addition and as previously discussed (Mukherjee et al., 2021), different subtypes may dynamically appear under stress or pathological conditions, and specific expression patterns may also depend on the niche environment (fetal liver vs bone marrow vs spleen (Gosselin et al., 2014; Lavin et al., 2014)) and modified by neural signaling in the bone marrow (Mendez-Ferrer et al., 2020). These may give rise to morphologically distinct classes of islands as observed by electron microscopy (Yeo et al., 2016).

In this context, one should avoid confusion between use of a protein as a marker versus its functional requirement for the cell. The suggestion that Vcam1 expression in macrophage is not required for erythroblastic island formation (Wei et al., 2019) does not negate its usefulness as a selection marker of a subset of islands. As another example, deletion of the widely-used F4/80 enrichment marker has no effect on macrophage viability/function (Schaller et al., 2002).

An additional challenge in the field has been the concern that pieces of macrophage can contaminate other cell types and have a confounding effect on conclusions from mRNA expression analysis. Contamination is apparent when analyzing flow-sorted single cells by image stream, where punctate expression from macrophage remnants attached at the cell surface are visible (Levesque et al., 2021). However, the extensive efforts to morphologically verify sorted single cell isolates, the inclusion of small peptides during isolation that disrupt erythroid/macrophage interactions, the verification of homogeneous distribution of marker expression in single cell images, and the clear segregation of cell surface expression used in the recent studies (Li et al., 2019; Mukherjee et al., 2021; Zhang et al., 2021) alleviate contamination concerns.

It is hoped the single cell studies provide foundation for breakthroughs in understanding heterogeneity and function by validation of FACS-based isolation methods for various subpopulations of F4/80+ macrophages. However, some outstanding questions remain:

### Developmental Onset of Critical Cell Types

There remains considerable ambiguity regarding what inherent cellular properties or external factors drive EBI niche formation by macrophages. In this context, recent studies implicating PPARγ in EBI development have opened up a window into the potential non-lipid-dependent role of this transcription factor in the adult bone marrow and spleen, perhaps by its modulation of macrophage subset lineage decisions and establishing identity (Okreglicka et al., 2021). Additionally, structural proteins such as tropomodulin3 are implicated in both erythroid and macrophage coordinate function in the EBI (Sui et al., 2014). However, in essence we know little about developmental origins of macrophages destined to form the EBI niche, and it appears from our single cell studies that there might be distinct populations of such macrophages likely originating from alternative sources. It is exciting to note that these subpopulations also express unique transcription factors, and this opens up multiple avenues of exploration (Figure 5). One interesting approach would be to examine how EKLF expression in these subpopulations is initiated and regulated. Further, one must determine how EKLF function in macrophages differs from its erythroid counterpart; since EKLF has a conserved DNA binding sequence (5′CCMCRCCCN; (Tallack et al., 2010; Gillinder et al., 2017; Kulczynska et al., 2020)), what prevents EKLF binding to erythroid targets and driving the erythroid gene program in macrophages? One likelihood is that EKLF has separate molecular interactions with transcription cofactors leading to distinct chromatin dynamics at EKLF targets in erythroid cells and macrophages, perhaps determined by its cell- or stage-specific post-translational modification status (discussed in Yien and Bieker (2013)).

### Alternate Island Populations

We have not addressed other cell types that may also be playing a role in the island niche. For example, the Kalfa lab analyzed single cells derived from isolated whole erythroblastic islands (Seu et al., 2018). Their data support the idea that non-F4/80 cells (ie, F4/80-Ly6G+ granulocytes) may play a role in a separate subset of island function, particularly as related to myelopoiesis following anemia of inflammation, leading to a dynamic antagonistic balance between granulopoiesis and erythropoiesis, a notion supported by the dramatic effects of G-CSF treatment on suppression of erythropoiesis (Jacobsen et al., 2014; Jacobsen et al., 2015). These data are consistent with imaging multi-color fluorescence analyses showing that F4/80, VCAM1, and CD169 (Siglec1), but not CD11b (Itgam) or Ly6G, are expressed in erythroblastic islands, while at the same time, the CD11b+Ly6G+F4/80- cells can be found “at the periphery” of a subset of these aggregates (Tay et al., 2020). The dynamic balance between these populations in the bone marrow versus the spleen, and whether they reside in the same ‘niche’ or are separate entities, will be quite interesting to quantify and delineate.

### Role of EKLF Mutations in Erythroblastic Island Macrophage Alterations

Two dominant monoallelic mutations in mammalian EKLF are causative for hematologic deficiencies: the mouse Nan mutation (E339D) leads to hemolytic neonatal anemia, and the human CDA mutation (E325K) causes congenital dyserythropoietic anemia (CDA) type IV (reviewed in Kulczynska-Figurny et al. (2020)). Although numerous other monoallelic mutations have been identified that lead to haploinsufficiency (Waye and Eng, 2015; Perkins et al., 2016), these have mild phenotypic effects. However, inheritance of two altered alleles can lead to compound heterozygosity and effects on blood cell production and properties, leading to anemia (Perkins et al., 2016; Tangsricharoen et al., 2021; Xu et al., 2021). These analyses have completely focused on effect on the red cell; however, it is easily conceivable that the island macrophage is also affected, particularly as the Nan and CDA mutations alter EKLF DNA recognition properties that lead to neomorphic expression changes and systemic effects (Kulczynska-Figurny et al., 2020).

### Relevance to Human Biology

Human macrophage also exhibit an extensive level of heterogeneity during development (Bian et al., 2020). However, many of the same subtype expression patterns described here for the mouse are also apparent from single cell analysis of human fetal liver cells, particularly within the “erythroblastic island macrophage” cluster (Popescu et al., 2019). In addition, a role for EKLF in proper programming of macrophage for island function is suggested by gain-of-function studies in macrophage derived from human iPS cells (Lopez-Yrigoyen et al., 2019). There remains a need to increase the expansion capability of erythroid cells in culture for their clinical use (Migliaccio et al., 2012; Heshusius et al., 2019; Olivier et al., 2019; Deleschaux et al., 2020; Pellegrin et al., 2021), and identification of the relevant macrophage subtype can inform approaches to overcome heterogeneity and utilize the optimal cells to aid efficient *in vitro* erythropoiesis (Rhodes et al., 2008; Hom et al., 2015; May and Forrester, 2020). Indeed, human island macrophage function can be recapitulated in culture by inclusion of lipids and by glucocorticoid receptor activation (Falchi et al., 2015; Heideveld et al., 2018).

## Final Comments

Establishing the necessity of EBI macrophage function for *in vivo* erythropoiesis remains challenging since contingency mechanisms have evolved to compensate for their loss. Future work in this area must aim to deplete not only EBI macrophages but also monocytes, but that can lead to other complications with immune function, unrelated to erythropoiesis. Still, the exact physiological role of EBIs in erythropoiesis at different stages of development, in different hematopoietic tissues, and in the context of diseases such as anemia and dysplasia, remains to be extensively characterized. Overall, the study of EBI macrophages is at an exciting juncture where the progress that has been made in gene expression studies can be extended into various erythropoietic tissues and conditions, and used as a guide for developing hypotheses to characterize their molecular nature and developmental origins.

## References

[B1] Alam M. Z. Devalaraja S. Haldar M. (2017). The Heme Connection: Linking Erythrocytes and Macrophage Biology. Front. Immunol. 8, 33. 10.3389/fimmu.2017.00033 28167947 PMC5256077

[B2] Bernard J. (1991). The Erythroblastic Island: Past and Future. Blood Cells 17 (1), 5–4. 2018860

[B3] Bessis M. (1958). Erythroblastic Island, Functional unity of Bone Marrow. Rev. Hematol. 13 (1), 8–11. 10.1182/blood.v13.4.410.410 13555228

[B4] Bian Z. Gong Y. Huang T. Lee C. Z. W. Bian L. Bai Z. (2020). Deciphering Human Macrophage Development at Single-Cell Resolution. Nature 582 (7813), 571–576. 10.1038/s41586-020-2316-7 32499656

[B5] Bieker J. J. (2010). Putting a finger on the Switch. Nat. Genet. 42 (9), 733–734. 10.1038/ng0910-733 20802474 PMC3234686

[B6] Buesche G. Teoman H. Giagounidis A. Gohring G. Schlegelberger B. Ganser A. (2016). Impaired Formation of Erythroblastic Islands Is Associated with Erythroid Failure and Poor Prognosis in a Significant Proportion of Patients with Myelodysplastic Syndromes. Haematologica 101 (5), e177–e181. 10.3324/haematol.2015.129015 26944473 PMC5004355

[B7] Cao S. Liu J. Song L. Ma X. (2005). The Protooncogene C-Maf Is an Essential Transcription Factor for IL-10 Gene Expression in Macrophages. J. Immunol. 174 (6), 3484–3492. 10.4049/jimmunol.174.6.3484 15749884 PMC2955976

[B8] Chasis J. A. Mohandas N. (2008). Erythroblastic Islands: Niches for Erythropoiesis. Blood 112 (3), 470–478. 10.1182/blood-2008-03-077883 18650462 PMC2481536

[B9] Chen Y. Xiang J. Qian F. Diwakar B. T. Ruan B. Hao S. (2020). Epo Receptor Signaling in Macrophages Alters the Splenic Niche to Promote Erythroid Differentiation. Blood 136 (2), 235–246. 10.1182/blood.2019003480 32350523 PMC7357191

[B10] Chow A. Huggins M. Ahmed J. Hashimoto D. Lucas D. Kunisaki Y. (2013). CD169+ Macrophages Provide a Niche Promoting Erythropoiesis under Homeostasis and Stress. Nat. Med. 19 (4), 4293057–4293436. 10.1038/nm.3057 PMC3983996 23502962

[B11] de Back D. Z. Kostova E. B. van Kraaij M. van den Berg T. K. van Bruggen R. (2014). Of Macrophages and Red Blood Cells; A Complex Love story. Front. Physiol. 5, 9. 10.3389/fphys.2014.00009 24523696 PMC3906564

[B12] Deleschaux C. Moras M. Lefevre S. D. Ostuni M. A. (2020). An Overview of Different Strategies to Recreate the Physiological Environment in Experimental Erythropoiesis. Int. J. Mol. Sci. 21 (15), 5263. 10.3390/ijms21155263 PMC7432157 32722249

[B13] Eaton S. A. Funnell A. P. W. Sue N. Nicholas H. Pearson R. C. M. Crossley M. (2008). A Network of Krüppel-like Factors (Klfs). J. Biol. Chem. 283 (40), 26937–26947. 10.1074/jbc.m804831200 18687676 PMC2556010

[B14] Falchi M. Varricchio L. Martelli F. Masiello F. Federici G. Zingariello M. (2015). Dexamethasone Targeted Directly to Macrophages Induces Macrophage Niches that Promote Erythroid Expansion. Haematologica 100 (2), 178–187. 10.3324/haematol.2014.114405 25533803 PMC4803138

[B15] Fraser S. T. Midwinter R. G. Coupland L. A. Kong S. Berger B. S. Yeo J. H. (2015). Heme Oxygenase-1 Deficiency Alters Erythroblastic Island Formation, Steady-State Erythropoiesis and Red Blood Cell Lifespan in Mice. Haematologica 100 (5), 601–610. 10.3324/haematol.2014.116368 25682599 PMC4420209

[B16] Funnell A. P. W. Maloney C. A. Thompson L. J. Keys J. Tallack M. Perkins A. C. (2007). Erythroid Krüppel-like Factor Directly Activates the Basic Krüppel-like Factor Gene in Erythroid Cells. Mol. Cell Biol 27 (7), 2777–2790. 10.1128/mcb.01658-06 17283065 PMC1899893

[B17] Ganz T. (2016). Macrophages and Iron Metabolism. Microbiol. Spectr. 4 (5), MCHD-0037-2016. 10.1128/microbiolspec.MCHD-0037-2016 27763254

[B18] Gautier E. L. Shay T. Shay T. Miller J. Greter M. Jakubzick C. (2012). Gene-expression Profiles and Transcriptional Regulatory Pathways that Underlie the Identity and Diversity of Mouse Tissue Macrophages. Nat. Immunol. 13 (11), 1118–1128. 10.1038/ni.2419 23023392 PMC3558276

[B19] Gillinder K. R. Ilsley M. D. Nébor D. Sachidanandam R. Lajoie M. Magor G. W. (2017). Promiscuous DNA-Binding of a Mutant Zinc finger Protein Corrupts the Transcriptome and Diminishes Cell Viability. Nucleic Acids Res. 45 (3), 1130–1143. 10.1093/nar/gkw1014 28180284 PMC5388391

[B20] Gnanapragasam M. N. Bieker J. J. (2017). Orchestration of Late Events in Erythropoiesis by KLF1/EKLF. Curr. Opin. Hematol. 24 (3), 183–190. 10.1097/MOH.0000000000000327 28157724 PMC5523457

[B21] Gosselin D. Link V. M. Romanoski C. E. Fonseca G. J. Eichenfield D. Z. Spann N. J. (2014). Environment Drives Selection and Function of Enhancers Controlling Tissue-specific Macrophage Identities. Cell 159 (6), 1327–1340. 10.1016/j.cell.2014.11.023 25480297 PMC4364385

[B22] Haldar M. Kohyama M. So A. Y.-L. Kc W. Wu X. Briseño C. G. (2014). Heme-mediated SPI-C Induction Promotes Monocyte Differentiation Into Iron-Recycling Macrophages. Cell 156 (6), 1223–1234. 10.1016/j.cell.2014.01.069 24630724 PMC4010949

[B23] Heideveld E. Hampton-O’Neil L. A. Cross S. J. van Alphen F. P. J. van den Biggelaar M. Toye A. M. (2018). Glucocorticoids Induce Differentiation of Monocytes towards Macrophages that Share Functional and Phenotypical Aspects with Erythroblastic Island Macrophages. Haematologica 103 (3), 395–405. 10.3324/haematol.2017.179341 29284682 PMC5830394

[B24] Heshusius S. Heideveld E. Burger P. Thiel-Valkhof M. Sellink E. Varga E. (2019). Large-scale *In Vitro* Production of Red Blood Cells from Human Peripheral Blood Mononuclear Cells. Blood Adv. 3 (21), 3337–3350. 10.1182/bloodadvances.2019000689 31698463 PMC6855111

[B25] Hom J. Dulmovits B. M. Mohandas N. Blanc L. (2015). The Erythroblastic Island as an Emerging Paradigm in the Anemia of Inflammation. Immunol. Res. 63 (1–3), 75–89. 10.1007/s12026-015-8697-2 26376896 PMC4651743

[B26] Jacobsen R. N. Forristal C. E. Raggatt L. J. Nowlan B. Barbier V. Kaur S. (2014). Mobilization with Granulocyte Colony-Stimulating Factor Blocks Medullar Erythropoiesis by Depleting F4/80+VCAM1+CD169+ER-HR3+Ly6G+ Erythroid Island Macrophages in the Mouse. Exp. Hematol. 42 (7), 547–5618. 10.1016/j.exphem.2014.03.009 24721610

[B27] Jacobsen R. N. Perkins A. C. Levesque J.-P. (2015). Macrophages and Regulation of Erythropoiesis. Curr. Opin. Hematol. 22 (3), 212–219. 10.1097/MOH.0000000000000131 25693142

[B28] Klei T. R. L. Meinderts S. M. van den Berg T. K. van Bruggen R. (2017). From the Cradle to the Grave: The Role of Macrophages in Erythropoiesis and Erythrophagocytosis. Front. Immunol. 8, 73. 10.3389/fimmu.2017.00073 28210260 PMC5288342

[B29] Kohyama M. Ise W. Edelson B. T. Wilker P. R. Hildner K. Mejia C. (2009). Role for Spi-C in the Development of Red Pulp Macrophages and Splenic Iron Homeostasis. Nature 457 (7227), 318–321. 10.1038/nature07472 19037245 PMC2756102

[B30] Korolnek T. Hamza I. (2015). Macrophages and Iron Trafficking at the Birth and Death of Red Cells. Blood 125 (19), 2893–2897. 10.1182/blood-2014-12-567776 25778532 PMC4424413

[B31] Koury M. J. (2014). Abnormal Erythropoiesis and the Pathophysiology of Chronic Anemia. Blood Rev. 28 (2), 49–66. 10.1016/j.blre.2014.01.002 24560123

[B32] Kulczynska K. Bieker J. J. Siatecka M. (2020). A Krüppel-like Factor 1(KLF1) Mutation Associated with Severe Congenital Dyserythropoietic Anemia Alters its DNA-Binding Specificity. Mol. Cell Biol. 40 (5), e00444–19. 10.1128/MCB.00444-19 31818881 PMC7020642

[B33] Kulczynska-Figurny K. Bieker J. J. Siatecka M. (2020). Severe Anemia Caused by Dominant Mutations in Krüppel-like Factor 1 (KLF1). Mutat. Res. Rev. Mutat. Res. 786, 108336. 10.1016/j.mrrev.2020.108336 33339573 PMC10199782

[B34] Kusakabe M. Hasegawa K. Hamada M. Nakamura M. Ohsumi T. Suzuki H. (2011). c-Maf Plays a Crucial Role for the Definitive Erythropoiesis that Accompanies Erythroblastic Island Formation in the Fetal Liver. Blood 118 (5), 1374–1385. 10.1182/blood-2010-08-300400 21628412

[B35] Lavin Y. Winter D. Blecher-Gonen R. David E. Keren-Shaul H. Merad M. (201401449). Tissue-resident Macrophage Enhancer Landscapes are Shaped by the Local Microenvironment. Cell 159 (614), 1312–13264. 10.1016/j.cell.2014.11.018 PMC4437213 25480296

[B36] Levesque J.-P. Summers K. M. Bisht K. Millard S. M. Winkler I. G. Pettit A. R. (2021). Macrophages Form Erythropoietic Niches and Regulate Iron Homeostasis to Adapt Erythropoiesis in Response to Infections and Inflammation. Exp. Hematol., S0301-472x(21)00291-5 [Epub ahead of print]. 10.1016/j.exphem.2021.08.011 34500024

[B37] Li W. Guo R. Song Y. Jiang Z. (2020). Erythroblastic Island Macrophages Shape Normal Erythropoiesis and Drive Associated Disorders in Erythroid Hematopoietic Diseases. Front. Cell Dev. Biol. 8, 613885. 10.3389/fcell.2020.613885 33644032 PMC7907436

[B38] Li W. Wang Y. Zhao H. Zhang H. Xu Y. Wang S. (2019). Identification and Transcriptome Analysis of Erythroblastic Island Macrophages. Blood 134, 480–491. 10.1182/blood.2019000430 31101625 PMC6676133

[B39] Liao C. Prabhu K. S. Paulson R. F. (2018). Monocyte-derived Macrophages Expand the Murine Stress Erythropoietic Niche during the Recovery from Anemia. Blood 132 (24), 2580–2593. 10.1182/blood-2018-06-856831 30322871 PMC6293871

[B40] Lifshitz L. Tabak G. Gassmann M. Mittelman M. Neumann D. (2010). Macrophages as Novel Target Cells for Erythropoietin. Haematologica 95 (11), 1823–1831. 10.3324/haematol.2010.025015 20595096 PMC2966903

[B41] Link V. M. Duttke S. H. Chun H. B. Holtman I. R. Westin E. Hoeksema M. A. (2018). Analysis of Genetically Diverse Macrophages Reveals Local and Domain-wide Mechanisms that Control Transcription Factor Binding and Function. Cell 173 (7), 1796–1809. 10.1016/j.cell.2018.04.018 29779944 PMC6003872

[B42] Lohmann F. Bieker J. J. (2008). Activation of Eklf Expression during Hematopoiesis by Gata2 and Smad5 Prior to Erythroid Commitment. Development 135 (12), 2071–2082. 10.1242/dev.018200 18448565

[B43] Lopez-Yrigoyen M. Yang C.-T. Fidanza A. Cassetta L. Taylor A. H. McCahill A. (2019). Genetic Programming of Macrophages Generates an *In Vitro* Model for the Human Erythroid Island Niche. Nat. Commun. 10 (1), 881. 10.1038/s41467-019-08705-0 30787325 PMC6382809

[B44] Luo B. Gan W. Liu Z. Shen Z. Wang J. Shi R. (2016). Erythropoeitin Signaling in Macrophages Promotes Dying Cell Clearance and Immune Tolerance. Immunity 44 (2), 287–302. 10.1016/j.immuni.2016.01.002 26872696

[B45] Manwani D. Bieker J. J. (2008). Chapter 2 the Erythroblastic Island. Curr. Top. Dev. Biol. 82, 23–53. 10.1016/s0070-2153(07)00002-6 18282516 PMC3234703

[B46] Mass E. Ballesteros I. Farlik M. Halbritter F. Günther P. Crozet L. (2016). Specification of Tissue-Resident Macrophages During Organogenesis. Science 353 (6304), aaf4238. 10.1126/science.aaf4238 27492475 PMC5066309

[B47] May A. Forrester L. M. (2020). The Erythroblastic Island Niche: Modeling in Health, Stress, and Disease. Exp. Hematol. 91, 10–21. 10.1016/j.exphem.2020.09.185 32910996

[B48] Méndez-Ferrer S. Bonnet D. Steensma D. P. Hasserjian R. P. Ghobrial I. M. Gribben J. G. (2020). Bone Marrow Niches in Haematological Malignancies. Nat. Rev. Cancer 20 (5), 285–298. 10.1038/s41568-020-0245-2 32112045 PMC9912977

[B49] Migliaccio A. R. Whitsett C. Papayannopoulou T. Sadelain M. (2012). The Potential of Stem Cells as an *In Vitro* Source of Red Blood Cells for Transfusion. Cell Stem Cell 10 (2), 115–119. 10.1016/j.stem.2012.01.001 22305561 PMC3676433

[B50] Mohandas N. Prenant M. (1978). Three-Dimensional Model of Bone Marrow. Blood 51 (4), 633–643. 10.1182/blood.v51.4.633.633 630113

[B51] Mukherjee K. Xue L. Planutis A. Gnanapragasam M. N. Chess A. Bieker J. J. (2021). EKLF/KLF1 Expression Defines a Unique Macrophage Subset during Mouse Erythropoiesis. Elife 10. 10.7554/eLife.61070 PMC7932694 33570494

[B52] Nakamura M. Hamada M. Hasegawa K. Kusakabe M. Suzuki H. Greaves D. R. (2009). c-Maf is Essential for the F4/80 Expression in Macrophages *In Vivo* . Gene 445 (1-2), 66–72. 10.1016/j.gene.2009.06.003 19539733

[B53] Okreglicka K. Iten I. Pohlmeier L. Onder L. Feng Q. Kurrer M. (2021). PPARγ Is Essential for the Development of Bone Marrow Erythroblastic Island Macrophages and Splenic Red Pulp Macrophages. J. Exp. Med. 218 (5). 10.1084/jem.20191314 PMC8006858 33765133

[B54] Olivier E. N. Zhang S. Yan Z. Suzuka S. Roberts K. Wang K. (2019). PSC-RED and MNC-RED: Albumin-free and Low-Transferrin Robust Erythroid Differentiation Protocols to Produce Human Enucleated Red Blood Cells. Exp. Hematol. 75, 31–52. 10.1016/j.exphem.2019.05.006 31176681

[B55] Paulson R. F. (2019). Epo Receptor marks the Spot. Blood 134 (5), 413134–413414. 10.1182/blood.2019001581 PMC6676130 31371394

[B56] Pellegrin S. Severn C. E. Toye A. M. (2021). Towards Manufactured Red Blood Cells for the Treatment of Inherited Anemia. Haematologica 106, 2304–2311. 10.3324/haematol.2020.268847 34042406 PMC8409035

[B57] Perkins A. Xu X. Higgs D. R. Patrinos G. P. Arnaud L. Bieker J. J. (2016). Krüppeling Erythropoiesis: an Unexpected Broad Spectrum of Human Red Blood Cell Disorders Due to KLF1 Variants. Blood 127 (15), 1856–1862. 10.1182/blood-2016-01-694331 26903544 PMC4832505

[B58] Pilon A. M. Arcasoy M. O. Dressman H. K. Vayda S. E. Maksimova Y. D. Sangerman J. I. (2008). Failure of Terminal Erythroid Differentiation in EKLF-Deficient Mice Is Associated with Cell Cycle Perturbation and Reduced Expression of E2F2. Mol. Cell Biol. 28 (24), 7394–7401. 10.1128/mcb.01087-08 18852285 PMC2593440

[B59] Popescu D.-M. Botting R. A. Stephenson E. Green K. Webb S. Jardine L. (2019). Decoding Human Fetal Liver Haematopoiesis. Nature 574 (7778), 365–371. 10.1038/s41586-019-1652-y 31597962 PMC6861135

[B60] Porcu S. Manchinu M. F. Marongiu M. F. Sogos V. Poddie D. Asunis I. (2011). Klf1 Affects DNase II-Alpha Expression in the central Macrophage of a Fetal Liver Erythroblastic Island: A Non-Cell-Autonomous Role in Definitive Erythropoiesis. Mol. Cell Biol. 31 (19), 4144–4154. 10.1128/mcb.05532-11 21807894 PMC3187365

[B61] Ramos P. Casu C. Gardenghi S. Breda L. Crielaard B. J. Guy E. (2013). Macrophages Support Pathological Erythropoiesis in Polycythemia Vera and β-Thalassemia. Nat. Med. 19 (4), 437–445. 10.1038/nm.3126 23502961 PMC3618568

[B62] Rhodes M. M. Kopsombut P. Bondurant M. C. Price J. O. Koury M. J. (2008). Adherence to Macrophages in Erythroblastic Islands Enhances Erythroblast Proliferation and Increases Erythrocyte Production by a Different Mechanism Than Erythropoietin. Blood 111 (3), 1700–1708. 10.1182/blood-2007-06-098178 17993612 PMC2214751

[B63] Sadahira Y. Yasuda T. Kimoto T. (1991). Regulation of Forssman Antigen Expression during Maturation of Mouse Stromal Macrophages in Haematopoietic Foci. Immunology 73 (4), 498–504. 1916901 PMC1384583

[B64] Sadahira Y. Yasuda T. Yoshino T. Manabe T. Takeishi T. Kobayashi Y. (2000). Impaired Splenic Erythropoiesis in Phlebotomized Mice Injected with CL2MDP-Liposome: An Experimental Model for Studying the Role of Stromal Macrophages in Erythropoiesis. J. Leukoc. Biol. 68 (4), 464–470. 11037966

[B65] Sadahira Y. Yoshino T. Monobe Y. (1995). Very Late Activation Antigen 4-vascular Cell Adhesion Molecule 1 Interaction is Involved in the Formation of Erythroblastic Islands. J. Exp. Med. 181 (1), 411–415. 10.1084/jem.181.1.411 7528776 PMC2191848

[B66] Schaller E. Macfarlane A. J. Rupec R. A. Gordon S. McKnight A. J. Pfeffer K. (2002). Inactivation of the F4/80 Glycoprotein in the Mouse Germ Line. Mol. Cell Biol 22 (22), 8035–8043. 10.1128/MCB.22.22.8035-8043.2002 12391169 PMC134735

[B67] Seu K. G. Papoin J. Fessler R. Hom J. Huang G. Mohandas N. (2017). Unraveling Macrophage Heterogeneity in Erythroblastic Islands. Front. Immunol. 8, 1140. 10.3389/fimmu.2017.01140 28979259 PMC5611421

[B68] Seu K. G. Romano L. Papoin J. Muench E. D. Konstantinidis D. Olsson A. (2018). “The Erythro-Myeloblastic Island (EMBI): A Hematopoietic Niche Balancing Erythropoiesis and Myelopopoiesis,” in American Society for Hematology Annual Meeting, San Diego, CA, December 3, 2018 (Washington, DC: American Society of Hematology).

[B69] Siatecka M. Bieker J. J. (2011). The Multifunctional Role of EKLF/KLF1 during Erythropoiesis. Blood 118 (8), 2044–2054. 10.1182/blood-2011-03-331371 21613252 PMC3292426

[B70] Soni S. Bala S. Gwynn B. Sahr K. E. Peters L. L. Hanspal M. (2006). Absence of Erythroblast Macrophage Protein (Emp) Leads to Failure of Erythroblast Nuclear Extrusion. J. Biol. Chem. 281 (29), 20181–20189. 10.1074/jbc.m603226200 16707498

[B71] Sui Z. Nowak R. B. Bacconi A. Kim N. E. Liu H. Li J. (2014). Tropomodulin3-null Mice Are Embryonic Lethal with Anemia Due to Impaired Erythroid Terminal Differentiation in the Fetal Liver. Blood 123 (5), 758–767. 10.1182/blood-2013-03-492710 24159174 PMC3907761

[B72] Tallack M. R. Keys J. R. Humbert P. O. Perkins A. C. (2009). EKLF/KLF1 Controls Cell Cycle Entry via Direct Regulation of E2f2. J. Biol. Chem. 284 (31), 20966–20974. 10.1074/jbc.m109.006346 19457859 PMC2742862

[B73] Tallack M. R. Perkins A. C. (2010). KLF1 Directly Coordinates Almost All Aspects of Terminal Erythroid Differentiation. IUBMB Life 62 (12), 886–890. 10.1002/iub.404 21190291

[B74] Tallack M. R. Whitington T. Shan Yuen W. Wainwright E. N. Keys J. R. Gardiner B. B. (2010). A Global Role for KLF1 in Erythropoiesis Revealed by ChIP-Seq in Primary Erythroid Cells. Genome Res. 20 (8), 1052–1063. 10.1101/gr.106575.110 20508144 PMC2909569

[B75] Tangsricharoen T. Natesirinilkul R. Phusua A. Fanhchaksai K. Ittiwut C. Chetruengchai W. (2021). Severe Neonatal Haemolytic Anaemia Caused by Compound Heterozygous KLF1 Mutations: Report of Four Families and Literature Review. Br. J. Haematol. 194, 626–634. 10.1111/bjh.17616 34227100

[B76] Tay J. Bisht K. McGirr C. Millard S. M. Pettit A. R. Winkler I. G. (2020). Imaging Flow Cytometry Reveals that Granulocyte colony-stimulating Factor Treatment Causes Loss of Erythroblastic Islands in the Mouse Bone Marrow. Exp. Hematol. 82, 33–42. 10.1016/j.exphem.2020.02.003 32045657

[B77] Toda S. Segawa K. Nagata S. (2014). MerTK-mediated Engulfment of Pyrenocytes by central Macrophages in Erythroblastic Islands. Blood 123 (25), 3963–3971. 10.1182/blood-2014-01-547976 24659633

[B78] Ulyanova T. Phelps S. R. Papayannopoulou T. (2016). The Macrophage Contribution to Stress Erythropoiesis: When Less is Enough. Blood 128 (13), 1756–1765. 10.1182/blood-2016-05-714527 27543439 PMC5043129

[B79] Wang J. Hayashi Y. Yokota A. Xu Z. Zhang Y. Huang R. (2018). Expansion of EPOR-Negative Macrophages besides Erythroblasts by Elevated EPOR Signaling in Erythrocytosis Mouse Models. Haematologica 103 (1), 40–50. 10.3324/haematol.2017.172775 29051279 PMC5777189

[B80] Waye J. S. Eng B. (2015). Krüppel-like Factor 1: Hematologic Phenotypes Associated withKLF1gene Mutations. Int. J. Lab. Hem. 37 (Suppl. 1), 78–84. 10.1111/ijlh.12356 25976964

[B81] Wei Q. Boulais P. E. Zhang D. Pinho S. Tanaka M. Frenette P. S. (2019). Maea Expressed by Macrophages, but Not Erythroblasts, Maintains Postnatal Murine Bone Marrow Erythroblastic Islands. Blood 133 (11), 1222–1232. 10.1182/blood-2018-11-888180 30674470 PMC6418477

[B82] Xiang J. Wu D.-C. Chen Y. Paulson R. F. (2015). *In Vitro* Culture of Stress Erythroid Progenitors Identifies Distinct Progenitor Populations and Analogous Human Progenitors. Blood 125 (11), 1803–1812. 10.1182/blood-2014-07-591453 25608563 PMC4357585

[B83] Xu L. Zhu D. Zhang Y. Liang G. Liang M. Wei X. (2021). Compound Heterozygosity for KLF1 Mutations Causing Hemolytic Anemia in Children: A Case Report and Literature Review. Front. Genet. 12, 691461. 10.3389/fgene.2021.691461 34249106 PMC8267787

[B84] Xue L. Galdass M. Gnanapragasam M. N. Manwani D. Bieker J. J. (2014). Extrinsic and Intrinsic Control by EKLF (KLF1) Within a Specialized Erythroid Niche. Development 141 (11), 2245–2254. 10.1242/dev.103960 24866116 PMC4034424

[B85] Yeo J. H. Colonne C. K. Tasneem N. Cosgriff M. P. Fraser S. T. (2019a). The Iron Islands: Erythroblastic Islands and Iron Metabolism. Biochim. Biophys. Acta Gen. Subj. 1863 (2), 466–471. 10.1016/j.bbagen.2018.10.019 30468802

[B86] Yeo J. H. Lam Y. W. Fraser S. T. (2019b). Cellular Dynamics of Mammalian Red Blood Cell Production in the Erythroblastic Island Niche. Biophys. Rev. 11, 873–894. 10.1007/s12551-019-00579-2 31418139 PMC6874942

[B87] Yeo J. H. McAllan B. M. Fraser S. T. (2016). Scanning Electron Microscopy Reveals Two Distinct Classes of Erythroblastic Island Isolated from Adult Mammalian Bone Marrow. Microsc. Microanal. 22 (2), 368–378. 10.1017/S1431927616000155 26898901

[B88] Yien Y. Y. Bieker J. J. (2013). EKLF/KLF1, a Tissue-Restricted Integrator of Transcriptional Control, Chromatin Remodeling, and Lineage Determination. Mol. Cell Biol. 33 (1), 4–13. 10.1128/mcb.01058-12 23090966 PMC3536305

[B89] Zhang H. Wang S. Liu D. Gao C. Han Y. Guo X. (2021). EpoR-tdTomato-Cre Mice Enable Identification of EpoR Expression in Subsets of Tissue Macrophages and Hematopoietic Cells. Blood 2021011410. 10.1182/blood.2021011410 PMC8767788 34098576

[B90] Zhao L. Lei W. Deng C. Wu Z. Sun M. Jin Z. (2021). The Roles of Liver X Receptor α in Inflammation and Inflammation‐Associated Diseases. J. Cell Physiol. 236 (7), 4807–4828. 10.1002/jcp.30204 33305467

